# Determinants of antimicrobial resistance occurrence in animal‐based food, perceived by livestock farmers: A qualitative phenomenological study

**DOI:** 10.1002/hsr2.1160

**Published:** 2023-03-21

**Authors:** Razie Toghroli, Teamur Aghamolaei, Laleh Hassani, Hamid Sharifi, Maziar Jajarmi

**Affiliations:** ^1^ Social Determinants in Health Promotion Research Center, Hormozgan Health institute Hormozgan University of Medical Sciences Bandar Abbas Iran; ^2^ HIV/STI Surveillance Research Center, and WHO Collaborating Center for HIV Surveillance, Institute for Futures Studies in Health Kerman University of Medical Sciences Kerman Iran; ^3^ Department of Pathobiology, Faculty of Veterinary Medicine Shahid Bahonar University of Kerman Kerman Iran

**Keywords:** antimicrobial resistance, food safety, livestock farmer, qualitative research

## Abstract

**Background and Purpose of the Study:**

The determinants of antimicrobial resistance in human medicine have been copiously explored. On the other hand, the existing research in veterinary medicine and animal husbandry is in its infancy. The present qualitative study used the one‐health approach to explore farmers’ attitudes toward antimicrobial use and antimicrobial stewardship.

**Methodology:**

The present qualitative study was phenomenological in type. It was conducted in 2022 in the two cities of Kerman and Bandar Abbas in Iran. The data were collected through in‐depth interviews by semistructured interviews with 17 livestock, poultry, and aquatic animal breeders selected through purposive sampling. The interviews lasted between 35 and 65 min in the Farsi language. The data were analyzed using conventional qualitative content analysis and Colaizzi's seven‐step analysis technique.

**Results:**

The open coding was done in MAXQDA 10 and the results of data analysis were classified into five main themes and 17 subthemes subcategories. The main categories include personal determinants, contextual determinants, legal and regulatory determinants, social determinants, and economic determinants.

**Conclusion:**

Considering the increasing use of antibiotics among livestock farmers and breeders of animals used as human food, different measures such as educational, regulatory, social, and even cultural interventions may be able to control and prevent antimicrobial resistance.

## 1 INTRODUCTION

Antimicrobial resistance (AMR) is an interspecies and global issue whose solution requires a global health‐related approach that takes into account the health of animals, humans, and the environment altogether.^1^, ^2^, ^3^ Concerning medicine and human health, many researchers have investigated physicians’ attitudes toward antibiotic resistance and drug prescription.^4^ In addition, different interventions have been made to reduce AMR and inappropriate prescriptions.^5^, ^6^, ^7^ Both qualitative and quantitative research methods in medicine have been carefully and widely used and the findings have shown that the prescription of antibiotics by doctors depends on clinical and nonclinical factors such as demographic, social, and psychological stimuli.^8^, ^9^, ^10^, ^11^, ^12^, ^13^

However, in veterinary medicine, investigating AMR has been limited to experimental designs and fewer attempts have been made to explore factors related to veterinarians, ranchers, and farmers.^14^ Some research in the United Kingdom showed that even antimicrobial drugs prescribed only by veterinarians are often used by farmers in the absence of veterinarians.^15^, ^16^ It seems that livestock breeders do not care much about AMR and prioritize their therapeutic decisions^17^, ^18^ and may not be aware of the health risks and complications caused by AMR.^18^, ^19^ In addition, since the products of this group are a very important contribution to the food consumption of people in society and they can use antibiotics without supervision in livestock breeding. Therefore, it is important to consider the determinants of antibiotic consumption from the point of view of livestock farmers and farmers to identify and biological experience of these people in this field with the aim of improving AMR conditions.^20^, ^21^, ^22^, ^23^ In addition, many medical interventions such as surgeries, organ transplants, and the treatment of cancer, autoimmune diseases, or child infections strongly depend on the use of antibiotics.^24^ Beyond humans, there are many populations including livestock, pets, and aquatic animals whose survival and treatment of infectious diseases depend on the use of antibiotics, either as growth promoters or for treatment or prevention; however, in unnecessary use of antibiotics, we are likely to develop drug resistance. The worrying fact is the increased rate of consuming antibiotics in the world by more than 70% between 2000 and 2010. More than two tons of antibiotics are used worldwide every 10 min, often without any supervision or prescription.^25^ The improper, inappropriate, and excessive use of antibiotics increases AMR. The effectiveness of antibiotics against bacteria that cause infectious diseases will be reduced.^26^, ^27^ Probably, we are facing terrible conditions in Asian and African countries due to insufficient stewardship. The rate of AMR and its mortality rate in the world, except in Wales and England^28^ has not been quantitatively documented. AMR is a long‐term disease of infectious origin, putting animals and humans alike at risk of death.^29^

Most population‐based studies of AMR and medical decisions have mainly used questionnaires and mostly followed a quantitative approach. Although surveys may analyze factors that may influence medical decisions based on qualitative research, it is possible to explore why and how various factors influence people's decisions. Qualitative approaches allow participants to freely and deeply explore their experiences, and decisions and share thoughts. However, so far only a few qualitative studies have explored farmers’ and ranchers’ experience of antimicrobial treatments but only in limited and specific contexts.^30^, ^31^, ^32^ Very little research has been conducted on health promotion in social sciences regarding antimicrobial stewardship among veterinarians and ranchers.^33^, ^34^ Recognizing the important role of scientists in health promotion and social sciences in dealing with AMR can be effective.^35^ Also, considering the limited research on ranchers’ and villagers’ medical decisions, there are probably many gaps in understanding the current state of the factors underlying these decisions.

Exploring the experiences of livestock farmers and growers of foods of animal origin provides a deep understanding of their experience of infectious diseases in the livestock and the problems of using available antimicrobial drugs and developing some knowledge of and attitude toward AMR. Unraveling the hidden facts about the problem and thinking of preventive solutions can improve the health of a whole society. The problems facing each stakeholder in AMR are unique to each group, and the use of qualitative research to examine the experiences of these people will reveal more features.^32^ This study aims to fill the gaps using a qualitative approach to discover the lived experiences of livestock farmers in the use of antibiotics on the farm, or in livestock, poultry and aquatic breeding centers. It aims to explore their attitude toward AMR and how this attitude may or may not help antimicrobial stewardship. Considering the significant role of ranchers and producers of raw food of animal origin, their lived experience is explored in the present study.

## 2 MATERIALS AND METHODS

### 2.1 Participants and sampling

The present qualitative study adopted a phenomenological approach. Phenomenology is the study of lived experiences of people facing a special phenomenon. Phenomenology seeks to reveal meanings as they are lived in everyday life.^31^ The phenomenological approach was justified by the fact that, logically, individuals are the best source of describing feelings, experiences, and situations when expressing themselves in words. The present participants are 17 livestock farmers that have experience in using antibiotics in Kerman and Bandar Abbas cities in Iran in 2022, selected through purposive sampling. All participants were literate. We first selected a number of breeders and livestock farmers who visited the two main veterinary pharmacies in Kerman and Bandar Abbas to buy antibiotics. They were invited to participate in the study. Each interviewee suggested the next one be interviewed. The inclusion criteria were being a breeder of livestock, poultry, and aquatic animals, as well as having the experience of self‐administration of antibiotics in animals under their care and willingness to participate in the research. The interviews continued until data saturation.^36^ When no new code was extracted from an interview, the sampling was stopped and the data saturation occurred. All the interviewers and the interviewees were Persian speakers.

### 2.2 Data collection and procedure

The interview guide was developed for this study. The guide included two parts: the first part asked for demographic information, including sex, marital status, education, socioeconomic status, residence status and type of work, and so on. The second part included semistructured open‐ended questions that were pilot tested with a scientist experienced in qualitative research.

The interviews were held face‐to‐face. The research questions dealt with the experience of livestock, poultry, and aquatic breeders in using antibiotics for livestock. The interview was in Farsi language and followed an exploratory approach. The purpose of the semistructured interview was to allow the interviewees to talk about their experiences easily and make it easy for the participants to answer. With these in‐depth interviews, which used clear, general, and open‐ended questions, the researcher helped the participants to share their work and personal life experiences. The primary questions of the interview were:

What do you know about the causes of AMR in Iran?

Have you ever used antibiotics arbitrarily in your livestock? Why?

Explain your experience of buying and taking antibiotics arbitrarily.

What do you think is the reason for the widespread use of antibiotics in Iran's animal husbandry industry?

What is your suggestion to prevent this problem?

The questions were designed in such a way that all the participants could understand the meaning of the questions and it would not be difficult for them to understand. During the interview, a number of other questions were also asked as the follow‐up. The researcher met the interviewees in their working world and partly their personal life to fully access their experiences. The interviews were recorded upon the participants’ written and verbal consent. Immediately completing each interview, it was transcribed and coded. When each interview was analyzed, the next interview followed. In the case of any ambiguity in content, another interview was held to solve the ambiguities. The interviews lasted between 35 and 65 min, depending on the participant's interest in continuing the interview. Immediately after each interview, all the interviews were transcribed in Microsoft Word.

### 2.3 Rigor and trustworthiness

To estimate reliability, two methods were used, one the review of content by the participants themselves and the other the review by the field specialists. The interviews were recorded and then transcribed. The content was reviewed by several experts in health education and one expert in bacteriology. After analyzing the data, it was re‐examined by the qualitative researchers. The revised manuscripts were sent to the participants to confirm the findings obtained by the researcher.

Also, to check the reliability of data, the four criteria proposed by Lincoln and Goba were used. These included: validity, reliability, verifiability, and transferability.^37^ To evaluate and increase the validity of findings, the sampling continued until data saturation. The data transfer was ensured by providing a comprehensive and complete description of the topic to the participants, with a subsequent data collection and analysis. Also, an external review was done to increase the reliability of the findings. The verifiability of findings was increased through triangulation.^38^

### 2.4 Data analysis

Data collection and analysis were carried out simultaneously. After the initial analysis of each interview, the next was planned. Each transcript of the interview was read three times to identify the hidden concepts in the statements of the participants. After extracting the initial codes, the codes were clustered into categories. Codes that shared similar meanings were grouped together. This process of analysis continued until the emergence of the main and subthemes. To facilitate the analysis of the qualitative data, the MAXQDA‐10 was used and also the data were analyzed using Colaizzi seven‐step method a content analysis, as described below:

1. Reviewing important findings and empathizing with the participants to understand them better.
2. Extracting important sentences related to the topic of interest.
3. Assigning specific concepts to the extracted sentences (formulating the meanings).
4. Categorizing the related formulas and concepts.
5. Combining the results in the form of a comprehensive description of the topic of interest.
6. Formulating a general description of the topic of interest explicitly and clearly as the intrinsic quality of the phenomenon.
7. Returning the content of results to the participants to check the reliability.^39^

## 3 ETHICS CONSIDERATIONS

In the interviews, the researchers introduced themselves and also explained the purpose of the research. They attempted to create an amicable atmosphere for the interview. The participants were also ensured of the confidentiality of the information they provided, the anonymity of recorded conversations, and also why they were selected to be included in the study. They consented to the voice recording. The participants were free to withdraw from the interview at any time they requested.

## 4 RESULTS

The mean age of participants was 39.5 years. The minimum and maximum age was 26 and 53 years, respectively. Other participants’ demographic characteristics are summarized in Table 1.

**Table 1:** Demographic characteristics of the study population.

Variable	Group	*f*.	%
Sex	Male	11	65
	Female	6	35
Marital status	Married	12	70
	Single	5	30
Education	<Diploma	10	60
	Diploma‐bachelor's degree	5	30
	>Bachelor's degree	2	10
Socioeconomic status (SES)	Low	5	30
	Moderate	7	40
	High or very high	5	30
Residence	Urban	8	47
	Rural	9	53
Type of work	Poultry	7	40
	Livestock	6	35
	Aquatic	4	25

Five main themes and 17 subthemes were obtained from the results of the qualitative phenomenology (Table 2).

**Table 2:** Categories and subcategories of livestock breeders' lived experiences of antibiotic use in animal‐based food.

Theme	Subthemes
Personal factors	Low health literacy
	Personality traits
	Lack of perceived susceptibility
Contextual factors	Limited access and facilities
	Transportation issue and distance
Regulatory factors	Inefficient rules and regulations
	Lacking cooperation of public sectors with livestock breeders
	Delayed services
Social factors	Medical advertisement
	Influence of important others
	Low perceived significance in society
Economic factors	Tendency for more production
	Prevention of loss
	Medical discount
	High medical and lab costs
	The threat for other livestock
	Insurance issues

### 4.1 Category 1: Personal factors

Subcategories: low AMR‐related health literacy (sound knowledge of the nature of AMR, knowledge of AMR occurrence and prevention, sound knowledge of administration), personality traits (responsibility, impatience in the effectiveness of drugs), and lacking perceived susceptibility.

#### 4.1.1 Low AMR‐related health literacy

Many of the present participants had no knowledge of the nature of AMR. The few cases that had heard about AMR did not know how to prevent this problem and how to deal with it. Some others had insufficient knowledge of the appropriate use of antibiotics. Here are two relevant accounts from the interviews:

*“When I don't know what you are talking about, what can I do about it? This is the first time ever I have heard about it. What is resistance?” (Participant #15: poultry breeder)*

*“We administer all drugs as 2 cc of intramuscular shots, no matter what the drug is”. (Participant #6: goat breeder)*

#### 4.1.2 Personality traits (low responsibility, impatience in the effectiveness of drugs)

Among what the livestock farmers mentioned about their experience of using antimicrobial drugs, was the personality traits. For example, they do not feel really responsible for the occurrence of this health issue, and second, they rush to see the effect of the drugs and change the administration or use the drugs arbitrarily.

*“Since long ago, we used the drugs as we wished, and they worked. Now, we do the same and we face no problem”. (Participant #2: aquatic farmer)*

*“We cannot wait for the livestock to recover. A cow means a lot to us. When one drug does not work, we go for another. The only thing we care about is for the cow to get well”. (Participant #11: cattle breeder)*

#### 4.1.3 Lack of perceived susceptibility

Some participants did not perceive themselves at the risk of AMR and did not see it as a critical issue. See below:

*“I don't think what you are telling is really a threat to us” (Participant #6: goat breeder)*

### 4.2 Category 2: Contextual factors

Subcategories:

limited access and facilities, transportation issue, and distance.

#### 4.2.1 Limited access and facilities

Unavailability of diagnostic and therapeutic measures in rural areas was a major reason for the arbitrary use of antibiotics in some participants. Here are some excerpts:

*“We have almost nothing in the village, no vet and no prescription. So, we do such things by ourselves”. (Participant #13: cow and sheep breeder)*

*“At midnight, if the livestock gets sick, there is no car to carry it to a clinic. So, we tend to use the drugs we already have”. (Participant #4: sheep breeder)*

*“They should set up a vet clinic and drugstore in rural areas which are highly populated too”. (Participant #4: sheep breeder)*

#### 4.2.2 Transportation and distance issue

The lack of transportation facilities and distance from medical centers in rural areas were mentioned among the reasons for the arbitrary use of antibiotics. Here is an excerpt:

*“If we decide to come to the city and see a doctor to prescribe some medicine, the animal may die” (Participant #13: cow and sheep breeder)*

### 4.3 Category 3: Regulatory factors

Subcategories: inefficient rules, lacking cooperation of public sector and livestock breeders, limited facilities.

#### 4.3.1 Inefficient rules and regulations

The lack of efficient and effective rules and regulations was mentioned among the reasons for arbitrary antibiotic use by the participants. Here is a relevant account:

*“When I am to sell the fish, nobody asks me if I gave it the necessary drugs or not. There is no rule to stop me, so I do what I do now”. (Participant #1: aquatic animal breeder)*

#### 4.3.2 Lacking cooperation of the public sector with livestock breeders

Many interviewees complained about the absence of training courses and the need for learning. This is deemed possible only if the public sector cooperates with ranchers to provide such training opportunities. Here is an excerpt:

*“The public sector almost has nothing to offer us. If there is any training, it is only on diseases and not on medications and whether to prescribe a specific drug or not”. (Participant #3: sheep breeder)*

*Delayed services: As some participants raised, they had to often wait long for the vet's advice or the test results from a lab. Thus, they were tempted to administer drugs arbitrarily*.

*“If we take the poultry to a lab and wait to receive the result, half of the stock already dies. We have no choice but to use antibiotics until we receive the lab test result in two or three days”. (Participant #16: poultry breeder)*

### 4.4 Category 4: Social factors

Subcategories: Medical advertisement, the influence of important others, low perceived significance in society.

#### 4.4.1 Medical advertisement

Some advertisements of drugs such as their magical effect, high quality, and wide‐ranging effect can encourage breeders’ arbitrary use of drugs. See an excerpt:

*“Some drugs are so famous that when we come across them in a shop, we buy many of them and store them for a rainy day”. (Participant #8: sheep breeder)*

#### 4.4.2 Influence of important others

Many participants confessed they bought the antibiotics that were mostly recommended by others whom they trusted greatly in life.

*“We use some colleagues’ experience. For one, when the loss of the stock is high, we begin to use antibiotics as recommended by the more experienced fellows. So we do not waste the time”. (Participant #12: Poultry breeder)*

*Low perceived significance in the society: As some participants pinpointed, AMR does not matter to the society. See the following accounts*:

*“Who knows what AMR is?! It is of no use. People are so financially pressed that do not care about it at all”. (Participant #1: aquatic farmer)*

### 4.5 Category 5: Economic factors

Subcategories: Tendency for more production, prevention of loss, medical discount, high medical and lab costs, the threat for other livestock, and insurance issues.

#### 4.5.1 Tendency for more production

Some participants shared their experience of consuming antibiotics to increase production and livestock growth.

*“Through experience, we know that antibiotics help the livestock grow better and more. The growth of the livestock is more than when it is not on antibiotics”*. (Participant #11: cattle breeder)

#### 4.5.2 Prevention of loss

Many breeders tend to use antibiotics arbitrarily even when not prescribed to prevent livestock loss.

*“I have no choice. If I wait to see a vet, I'll lose all I have before I get the lab test result. So, I prefer self‐medication to prevent the loss” (Participant #16: Poultry breeder)*

*Medical discount: Sometimes, especially industrial livestock breeders, who often possess large livestock, welcome drugs that are on sale at particular times of the year. They may want to buy and store a load of the drugs, which they will use more for sure in future*.

*“At particular times of the year, some drugs are on sale. We buy a lot of them at the drugstore at a lower price. When our poultry is sick, we will benefit from a herd of 20 thousand”. (Participant #17: poultry breeder)*

#### 4.5.3 High medical and lab costs

Some participants mentioned such obstacles as the high diagnostic and medical costs for the antibiotic selfmedication.

*“Generally, vetrinary drugs are barely more than three or four. Why should we pay high costs to visit a doctor or take lab tests? We know what we are expected to known”. (Participant #1: aquatic breeder)*

#### 4.5.4 The threat for other livestock

The perceived threat of the spread of a disease from one livestock to another was among the experiences shared by the participants.

*“It is a threat to a whole livestock to get sick. The one or two sick animals should be separated from others to save others”. (Participant #9: sheep breeder)*

#### 4.5.5 Insurance issues

The inefficiency and inadequacy of the insurance system in livestock and poultry and compensation worries most livestock farmers about livestock losses. Therefore, even though the breeders insure their livestock, they are worried about the loss of their livestock and do not hesitate to make any efforts to prevent these losses.

*“After a loss, the insurance company brings a reason for not paying the compensation. Even if it decides to pay, the amount is so low that is hardly helpful. All our money will be gone. We cannot wait and do nothing. We can not take the risk. We go for self‐medication hoping to lower the losses”*. (Participant #17: poultry breeder)

## 5 LIMITATIONS AND STRENGTHS OF THE STUDY

One limitation of the present study was access to livestock farmers who had the time and willingness to speak. To overcome this limitation and reach the farmers, we went to places that livestock farmers often visit, such as veterinary pharmacies. At the end of each interview, the participants were thanked with a small gift. Another limitation was that this study was qualitative in type. It is not possible to generalize the findings, because the present findings can be different depending on personal, social, economic, and even contextual and geographical factors. The present findings can be used to control and prevent AMR, improve stewardship and plan to control and prevent antimicrobials. Future investigations should include interventions in light of the present findings to provide solutions to the relevant problems.

## 6 DISCUSSION

The present study's results showed that personal, contextual, legal, regulatory, social, and economic factors are decisive factors in occurrence of AMR in animal‐based food, as perceived by livestock farmers. Low health literacy was one subcategory of the personal factors. The in‐depth qualitative study showed that the farmers and breeders of raw materials of animal‐based food participating in this study had sufficient knowledge of the possible risks of AMR. They did not have antimicrobial stewardship. Therefore, it is necessary to implement regular and continuous training programs with the cooperation of some agricultural jihadi organizations, animal medicine, and health departments of universities to solve the existing problems and prevent diseases among livestock farmers. In line with the present findings, Golding et al. found that livestock farmers and breeders of animal‐based food were not sufficiently aware of the possible risks induced by AMR and their role as antimicrobial stewards. These factors prevent them from always acting in line with the ideal responsibilities when making decisions about antimicrobial treatment. Therefore, the lenience of the Department of Livestock Production and Extension and the absence of any training course to familiarize ranchers with the basics of using antimicrobial drugs keep ranchers unaware of new livestock inputs, increase the risk of production, and ultimately reduce interest in ranching jobs in the future. Livestock breeders face issues due to economic challenges, competitive industry drivers, contextual factors on the farm, and different personality traits. Their concerns about and fear of losing the animals if not treated on time and the subsequent financial damage lead them to self‐medication, even when they know antimicrobial drugs are not needed. Similar findings have also been reported in other studies of veterinary medicine and human medicine.^4^, ^14^, ^40^ Moreover, the unavailability of antimicrobial drugs and fear of the adverse effects sometimes lead to their inappropriate use. Not only livestock farmers but also vets, in uncertain conditions instead of ceasing the treatment, tend to over‐prescribe or use antimicrobial drugs.^4^, ^41^ Among the interpersonal and contextual subcategories, lacking access and transportation problems were also raised. Probably, the number of different livestock diseases, limited access to experienced vets and damages caused by inappropriate conditions of keeping livestock in barns and stables, improper distribution, and price stability of products in the market are among the major issues in managing traditional assemblies of livestock farmers. These can increase livestock farmers’ tendency to use antimicrobial drugs. In Cheruiyot et al.'s study^42^ and other similar works of research, factors such as poor veterinary services, absence of vets, unavailability of livestock medicine or high prices, and the lack of appropriate breeding centers and laboratories in the immediate work environment of ranchers were raised, similar to the present study. Therefore, the following are all somehow related to vet services: high medical costs, lack of medicines in the market, high rate of animal deaths caused by fake medicine, severe damage to animal husbandry units due to delayed diagnosis, distant veterinary medical centers, and the absence of vets. Moreover, many vets prefer to take care of dogs and cats in cities rather than rural areas. Veterinary services are actually one problem of the animal husbandry sector, which is an important factor that causes livestock farmers to use arbitrary antimicrobial drugs. Another factor from the ranchers’ point of view was the regulatory factor, with the subcategories of inefficient regulations, lacking cooperation of public sectors and livestock breeders, and limited facilities. Legal support, government support, and ensured purchase from ranchers can reduce the residues of ranchers using antimicrobial drugs for their livestock. The problems related to dealers, failure to fulfill the officials’ promises about solving livestock issues such as livestock grazing and lack of government support in giving loans and facilities with low interest to ranchers due to their vulnerability have created insecure conditions for ranchers. The results of many studies^43^, ^44^ were consistent with the present findings. Therefore, it is recommended to take certain measures such as eliminating mediators and dealers, guaranteeing the purchase of products from ranchers, issuing grazing licenses to unlicensed ranchers, giving loans and facilities of low profit, monitoring and following up on the issues facing ranchers by an executive and regulatory bodies. Also, the lack of the required facilities, welfare and communication facilities, lack of transportation, and health services were among the other issues pinpointed by the ranchers. It is suggested that the government provide more facilities for the livestock farmers’ welfare. Among the important determinants found in this study are social factors. One subcategory is the influence of important others and pharmaceutical advertisements. Ranchers’ acquaintance with elders and exemplary ranchers and technical and local leaders who can play an important role in removing the obstacles and problems in ranching can be effective by sharing the information required to enhance the impression of important others.^45^ To enhance the quality of communications between livestock farmers and promoters, it is recommended to provide educational courses and workshops for livestock farmers to improve their professional skills in dealing with livestock and the relevant diseases. Many pharmaceutical advertisements in the livestock industry have caused more demand than the needs of livestock farmers and are used to treat diseases or increase the growth of livestock. It can unintentionally increase the pharmaceutical residues in this category of food.^46^ Baghloui et al.^47^ found that out of the total number of carcasses, the amount of antibiotic injection in animal carcasses was 17.38%. Mahmoudi et al.^48^ Concluded that a total of 6.3% of the carcasses contained antibiotic residues. It seems due to the breeders’ deficient knowledge or to prevent any loss, many animals not recovering from a disease are transferred to the slaughterhouse, and any drug and antibiotics may be used to treat them. When companies hold exhibitions or sell their pharmaceutical products at a discount, which causes livestock farmers to buy more than they need. Thus, consumption increases too. Therefore, there is a need for balanced pharmaceutical advertisements. Among the economic subcategories was the insurance status, which is consistent with the findings reported by Mirzaei et al.^49^ and Ansari‐Renan et al.^43^ Livestock breeders were concerned about the insurance status, giving rise to issues such as the lacking supportive fund for breeders, strict insurance rules, and the low payment for damages to breeders. The reasons why the livestock farmers are not willing to insure their livestock and they are not satisfied with the insurance services are multi‐dimensional. Instances are the insurance company not paying compensation on time, the high insurance premium, the low estimated payment for compensation, and the high medical costs of diagnostic and lab measures. These have led to the ranchers’ dissatisfaction and unwillingness to insure the livestock. These problems can be partly reduced by supporting farmers and providing services such as livestock insurance and financial support for the livestock farmers.

## 7 CONCLUSION

The current findings show that the determinants of increasing AMR are alarmingly high from the point of view of livestock and poultry breeders. Based on the lived experience of these people from purchasing and consuming AMR in their livestock, a large number of stimulants that reinforce this abuse will cause serious health problems in society (both for animals and humans). Therefore, it is certain that the knowledge of these determinants can help the human and animal society and the health care system. Therefore, designing and implementing interventions based on the results of this study can help control antibiotic resistance in society.

## AUTHOR CONTRIBUTIONS

**Razie Toghroli**: Conceptualization; methodology; writing—original draft; writing—review and editing. **Teamur Aghamolaei**: Investigation; methodology; writing—original draft. **Laleh Hassani**: Conceptualization; investigation; supervision; writing—original draft. **Hamid Sharifi**: Data curation; investigation; resources. **Maziar Jajarmi**: Writing—original draft; writing—review and editing.

## CONFLICT OF INTEREST STATEMENT

The authors declare no conflicts of interest.

## ETHICS STATEMENT

This research was approved by the Ethics Committee of Hormozgan University of Medical Sciences (#IR.HUMS.REC.1400.207).

## TRANSPARENCY STATEMENT

The lead author Razie Toghroli affirms that this manuscript is an honest, accurate, and transparent account of the study being reported; that no important aspects of the study have been omitted; and that any discrepancies from the study as planned (and, if relevant, registered) have been explained.

## Data Availability

Data are available upon reasonable request to the corresponding author.
